# Who bought a gun during the COVID-19 pandemic in the United States?: Associations with QAnon beliefs, right-wing political attitudes, intimate partner violence, antisocial behavior, suicidality, and mental health and substance use problems

**DOI:** 10.1371/journal.pone.0290770

**Published:** 2023-08-29

**Authors:** Brian M. Hicks, Catherine Vitro, Elizabeth Johnson, Carter Sherman, Mary M. Heitzeg, C. Emily Durbin, Edelyn Verona

**Affiliations:** 1 Department of Psychiatry, University of Michigan, Ann Arbor, MI, United States of America; 2 Department of Psychology, Michigan State University, East Lansing, MI, United States of America; 3 Department of Psychology, University of South Florida, Tampa, Florida, United States of America; UCL: University College London, UNITED KINGDOM

## Abstract

There was a large spike in gun purchases and gun violence during the first year of the COVID-19 pandemic in the United States. We used an online U.S. national survey (*N* = 1036) to examine the characteristics of people who purchased a gun between March 2020 and October 2021 (*n* = 103) and compared them to non-gun owners (*n* = 763) and people who own a gun but did not purchase a gun during the COVID-19 pandemic (*n* = 170). Compared to non-gun owners, pandemic gun buyers were younger and more likely to be male, White race, and to affiliate with the Republican party. Compared to non-gun owners and pre-pandemic gun owners, pandemic gun buyers exhibited extreme elevations on a constellation of political (QAnon beliefs, pro-gun attitudes, Christian Nationalism, approval of former President Donald Trump, anti-vax beliefs, COVID-19 skepticism; mean Cohen’s *d* = 1.15), behavioral (intimate partner violence, antisocial behavior; mean *d* = 1.38), mental health (suicidality, depression, anxiety, substance use; mean *d* = 1.21), and personality (desire for power, belief in a dangerous world, low agreeableness, low conscientiousness; mean *d* = 0.95) characteristics. In contrast, pre-pandemic gun owners only endorsed more pro-gun attitudes (*d* = 0.67), lower approval of President Joe Biden (*d* = -0.41) and were more likely to be male and affiliate with the Republican party relative to non-gun owners. Pandemic gun buyers represent an extreme group in terms of political and psychological characteristics including several risk-factors for violence and self-harm.

## Introduction

The early stages of the COVID-19 pandemic introduced tremendous uncertainty and stress into people’s lives around the world that included major disruptions to social and economic life, in addition to the health risks associated with contracting a potentially life-threatening infectious disease. In the United States, the first year of the COVID-19 pandemic was also a period of social and political upheaval driven by the impact of heightened divisions among Americans. This upheaval included resistance to public health guidelines and government restrictions to curtail the spread of COVID-19, protests against police brutality that became the largest mass movement in U.S. history, controversies around the 2020 presidential election results (aka the Big Lie conspiracy theory of voter fraud), and the Insurrection of January 6th, 2021 when Trump supporters stormed the U.S. Capitol in a failed attempt to stop Congress from certifying the electoral college votes of the 2020 Presidential election.

Amidst this turmoil, there was a dramatic increase in domestic gun sales [1]. The estimated 7 million federal background check applications in the early part of the pandemic (March to July 2020) surpassed the rates recorded during other high gun-buying periods in recent history, including the months following the terrorist attacks of September 11, 2001, the election of Barack Obama in 2008, and the Sandy Hook school shooting in 2012 [1]. From March to July 2020, national surveys estimated that 6% of adults bought guns [2, 3], 34% of whom were new gun owners, for an estimated 6 million first time gun buyers [2].

Perhaps not coincidentally, there was an increase in shootings, murders, and firearm-related injuries beginning in the summer of 2020 that persisted through 2021 [4], although this increase in violence has yet to be conclusively tied to the rise in gun purchases. In 2020, guns were involved in 79% of homicides and 53% of suicides. Notably, there was a 35% increase in firearm-related homicides, 34% increase in firearm-related non-fatal injuries, and a 28% increase in all firearm-related deaths relative to 2019, though the firearm-related suicide rate was relatively stable [5, 6]. New York state recorded an increase in shootings that coincided with increases in gun purchases between February and April 2020 [7], and the city of Buffalo recorded an increase in shootings following the imposition of stay-at-home orders, relative to the period immediately prior to the pandemic [8]. Relative to previous years, trauma centers admitted more patients suffering from gunshot wounds from March to May 2020 [9], and an increase in firearm-related injuries involving young children in the first 6 months of the pandemic was correlated with the rise in new firearm ownership [10]. The rise in shootings and firearm-related injuries could be tied to the uncertainty and stress associated with lockdowns and the pandemic instead of the new gun sales [11], although the fact that more guns were in circulation could have been a contributing factor. Previous analyses show that state-level gun ownership rates are associated with firearm homicide rates [12], although causal relationships are not established. One way to examine whether pandemic-era gun purchases have the potential to lead to greater gun violence in the future involves an analysis of characteristics of the pandemic-era buyers. Specifically, we explored the extent to which pandemic era gun buyers exhibited characteristics associated with increased risk for gun violence.

### Characteristics of COVID-19 pandemic-era gun buyers

Who bought a gun during the COVID-19 pandemic? The stress and uncertainty of the pandemic and its associated social and political disruptions were likely a catalyst for new gun purchases. However, COVID-19 era gun buyers may also have been motivated by additional psychological factors, including factors that differentiate pandemic-era gun buyers from people who owned a gun prior to the pandemic but did not make new gun purchase during the pandemic (i.e., pre-COVID-19 pandemic gun owners). Gun owners, in general, differ from non-gun owners in systematic ways, including demographic factors (e.g., more likely to be male and White), political attitudes (e.g., conservative values and affiliation with the Republican party), and beliefs regarding self-protection and justifiable homicide [13]. To the extent that the pandemic-era gun purchases were disproportionately made by buyers at high-risk for violence or self-harm relative to non-gun owners and pre-pandemic gun owners, then more deleterious outcomes could follow from such purchases (e.g., suicides, shootings). For example, did people who bought a gun during the COVID-19 pandemic show risk factors (e.g., mental health and substance use problems) or have certain demographic characteristics (e.g., age, sex, race) associated with increased risk of gun violence or injuries?

In the first year of the pandemic, some cautioned that the adverse impact of pandemic-related strains and social isolation on mental health and suicide risk [14, 15] could lead to fatal consequences if vulnerable individuals had access to a firearm [16]. Indeed, persons who purchased or intended to purchase firearms during COVID-19 pandemic endorsed more mental health problems and suicidal ideation relative to non-gun owners and pre-pandemic gun owners who did not purchase a firearm during the COVID-19 pandemic [16, 17]. The same pattern was observed among persons with gun-shot injuries at Level I trauma centers across four states from March to May 2020. That is, these patients were more likely than in previous years to have experienced mental health and substance use problems in the past year [9]. Thus, one consideration is the extent to which pandemic-era gun buyers are more likely to experience psychological distress and suicidality, which would increase risk of future self-harm.

Pandemic-era gun purchases by persons who are at high-risk of domestic violence or aggression may also increase the risk of violence within the home if a gun is present [18–21]. The additional time that families and couples spend at home together during surges in COVID-19 cases could result in greater family conflict and domestic violence [22], which can have fatal consequences among vulnerable people who have access to guns. A review of 18 studies estimated a 5% increase in official reports of domestic violence following stay at home orders in 2020 [23]. Thus, an important gap to address is the extent to which pandemic-era gun buyers exhibit characteristics associated with domestic violence and aggression in general.

Besides risk factors associated with mental health problems and violence, there are likely to be differences in motivations and ideological worldviews among those who purchased guns during the pandemic and those who did not. There is preliminary evidence that pandemic-era gun buyers are more likely to associate guns with personal safety than non-gun owners [24–26], and enhanced fears of a dangerous world may have motivated gun owners to make their firearms more accessible during the early months of the pandemic [27, 28].

The extent to which safety motives are associated with political or ideological views is not yet known. Pandemic-era gun purchases have been linked to conservative or right-wing political views, support for former President Donald Trump, pro-gun attitudes, fear of government overreach, and disapproval of pandemic-related shutdowns [3, 24, 26, 29]. Other research, however, reported a bipartisan surge in gun-buying early in the pandemic [1, 26] and a disproportionate number of women and non-White racial and ethnic minorities purchasing guns for the first time during the pandemic [30]. Additional research then is needed to determine if pandemic-era gun buyers differ from non-gun owners and pre-pandemic gun owners in terms of their political attitudes.

### Present study

Most prior studies describing COVID-19 pandemic gun buyers have focused on purchases in the first few months of the pandemic and differences in demographic and COVID-19-related factors. We expanded on this work in two important ways. First, we compared COVID-19 gun buyers to non-gun owners and to persons who were gun owners prior to the pandemic, but who had not purchased a gun from March 2020—when the pandemic first began to disrupt social and economic activity in the United States—to October 2021. This allowed us to determine whether buying a gun during the period of the COVID-19 pandemic identified an important subgroup of gun owners relative to pre-pandemic gun owners and non-gun owners in general. We especially wanted to determine whether the pandemic-era gun buyers represented a subgroup of gun owners that exhibit risk factors associated with using a gun for the purpose of violence or self-harm.

Second, we compared COVID-19 pandemic gun buyers, pre-pandemic gun owners, and non-gun owners on a larger array of variables than prior studies to better describe their psychology. While our focus was on variables associated with violence to self and others (e.g., mental health, antisocial behavior, aggressive tendencies), we also compared the groups on demographic variables, political attitudes and conspiratorial beliefs, and COVID-19 related variables. Based on prior research associated with gun ownership, gun violence, and purchasing guns during the COVID-19 pandemic, we were able to make the following predictions, with some analyses exploratory in nature:

1. **Demographic variables.** We predicted COVID-19 pandemic gun buyers would have a younger mean age [31], and as is the case for gun owners in general, to be disproportionately male and White race relative to non-gun owners [32, 33].
2. **Prior history of violence.** Given the higher rates of gun violence that coincided with the increase in gun purchases, we predicted pandemic gun buyers would report higher rates of intimate partner violence and antisocial behavior, which are strong correlates of gun violence, than non-gun owners [34–36].
3. **Mental health and substance use problems.** Based on reports of higher rates of suicidality in pandemic era gun buyers [9, 16] and higher rates of substance use problems associated with gun violence [37], we anticipated more mental health (e.g., depression, anxiety, suicidality) and substance use problems among the pandemic gun buyers relative to the other groups.
4. **Personality.** Given the anticipated associations with violence, antisocial behavior, and mental health and substance use problems, we expected pandemic gun buyers to score higher on personality measures associated with aggression (low agreeableness), impulsivity (low conscientiousness), negative emotions, and a desire for more control and power than non-gun owners.
5. **Political attitudes.** We predicted pandemic gun buyers and pre-pandemic gun owners would have higher pro-gun attitudes than non-gun owners. We also predicted pandemic gun buyers would endorse more conservative or right-wing political beliefs including affiliation with the Republican party, greater approval of former U.S. President Donald Trump, and support for Christian nationalism than non-gun owners. These differences were expected given that pandemic-era gun sales coincided with a surge of political opposition to pandemic-related safety precautions and conspiratorial movements in support of former President Donald Trump and against the election of President Joe Biden. Pandemic gun buyers would also report more fears about dangers to their personal safety and property than non-gun owners. Further, these fears would extend to include beliefs in a dangerous and degrading society and endorsement of conspiracy theories such as QAnon.
6. **COVID-19 variables.** Given the anticipated associations with right-wing political attitudes (which are associated with less support for government mandates and personal mitigation strategies for COVID-19 [38]), we predicted that pandemic gun buyers would report a lower level of concern about the health risks of COVID-19 than non-gun owners.

## Methods

### Sample

Data were collected from September 16 through October 11, 2021, as part of the fifth wave of the COVID-19 Adjustment and Behaviors study [38] using an actively managed, double-opt-in research panel using Qualtrics XM survey software. Recruitment was designed to ascertain a sample consistent with major demographic characteristics of the United States adult population for age (12.8% 18–24; 17.7% 25–34; 16.7% 35–44; 17.7% 45–54; 16.4% 55–64; 18.8% +65), sex assigned at birth (51% female; 49% male), and race/ethnicity (61.9% non-Hispanic White; 12.3% non-Hispanic Black; 17.4% Hispanic; 5.3% Asian; 3.2% Other). Quotas were created for each variable and monitored while the survey was in the field. Respondents were recruited using a dashboard-style web page on the Qualtrics website and cellphone app where participants see a list of surveys that they have the option to participate in. Recruitment was also conducted through emails sent to established panel members within the Qualtrics database. In all recruitment methods, potential participants received information on the estimated length of the survey and compensation for completing it. Specific details about the survey content were not available until the participants opted-in to avoid self-selection bias. Upon opting into the study, participants read and provided an electronic signature on a consent form containing an overview of the survey contents. Participation was voluntary and anonymous as no individually identifying information was collected. Contact information for the research team was provided in case participants had questions about the survey. Qualtrics was paid a flat fee per completed survey and the compensation rate to participants was set by Qualtrics based on pre-determined rates for estimated time and complexity of the survey. The University of Michigan Medical School Institutional Review Board (IRB) reviewed the study design, and determined the study was exempt from further oversight (HUM00170909).

The survey for the current analyses was completed by 1050 respondents; 593 respondents had completed a prior survey before this wave, and 457 were new respondents. Data were manually checked, and 14 respondents were excluded due to inconsistent and illogical responses, resulting in a final sample size of 1036 respondents (526 men, 510 women). Single measures were excluded on a case-by-case basis if all other responses from that participant were within a plausible range of values. The demographic characteristics for this sample were as follows. The mean age was 47.5 years (SD = 16.0 years) with a range of 18 to 90 years old. The reported racial/ethnic identity was 62.0% non-Hispanic White (*n* = 642), 12.8% non-Hispanic Black (*n* = 133), 6.3% (*n* = 65) Asian, 1.6% (*n* = 17) other races and 17.3% (*n* = 179) identified as Hispanic. Participants resided in 47 states and the District of Columbia (no participants reported living in Maine, Montana, or Wyoming), and the state-level representation was similar to, but not the same as, the actual state-to-nation proportions. The median response time for completing the survey was 26.6 minutes.

### Measures

#### Gun ownership and purchases

Participants were asked, “Do you own a gun or rifle?”. If yes, participants were asked a series of follow-up questions regarding the type (handgun, rifle or long gun) and number of guns, whether they carried a firearm outside the home other than for hunting, if they had a state issued permit to carry a firearm, primary reason for owning a firearm, and how their firearm was stored. Finally, all participants were asked, “Have you purchased any guns since March 2020, which is roughly the start of the COVID-19 pandemic in the United States?”.

#### Demographics

Demographic characteristics used included age and sex (male, female), race, Hispanic ethnicity, annual household income, and educational attainment. We also assessed political party affiliation (Democratic, Republican, independent or unaffiliated, third party).

### Outcome variables

#### Political attitudes

We included questions and scales to assess a variety of attitudes and beliefs related to conventional political topics (e.g., Presidential approval) and more extreme conspiratorial beliefs (QAnon) which are described below:

#### Presidential and past presidential approval

We asked respondents to report their approval of President Biden’s job performance in general and past President Trump’s overall approval *(strongly approve*, *approve*, *somewhat approve*, *somewhat disapprove*, *disapprove*, *strongly disapprove)*.

#### Pro-gun attitudes

We used a four-item scale to assess attitudes about state regulations on the purchase and ownership of personal firearms and attitudes about gun ownership in general (e.g., *The best defense against a tyrannical government is a well-armed citizenry)* (α = .84 [38]).

#### Pro-police attitudes

We used a 5-item scale to assess perceptions of police misconduct (e.g., *Police are more concerned about exerting their authority than protecting citizens and enforcing laws*.*—reversed scored*) and racial bias in policing (e.g., *The police treat Black and White people equally*) (α = .79 [38]).

#### Christian nationalism

We used a six-item scale to assess Christian nationalism, the belief that policies of the United States federal government should reflect and support a particular form of Christian identity and culture (e.g., *The federal government should declare the United States a Christian nation*.) (α = .86 [39]).

#### Qanon

We used six items to develop a scale (α = .88) to assess attitudes toward QAnon (e.g., *How often do you think you can trust QAnon to provide accurate information*? [40]), and agreement with the most common QAnon political claims (e.g., *The government*, *media*, *and financial worlds in the U*.*S*. *are controlled by a group of Satan-worshipping pedophiles who run a global child sex trafficking operation* [41]).

#### Beliefs about COVID-19

We included questions and scales to assess COVID-19-related attitudes to examine associations between gun ownership groups and COVID-19 beliefs including:

#### Anti-vax beliefs

We used a four-item scale assessing general support for vaccinations to assess anti-vaccination (i.e., anti-vax) attitudes (e.g., *Vaccines are more dangerous than the diseases they are trying to prevent*.) (α = .91 [42]).

#### COVID-19 risk

We used a six-item scale to assess perceptions of risk for contracting or spreading the COVID-19 virus while engaging in common activities including dining at a restaurant indoors and outdoors, going to a bar indoors and outdoors, attending a large gathering indoors (more than 15 people) and outdoors (more than 25 people) (α = .92).

#### COVID-19 skepticism

We used a four-item scale that asked participants to rate their level of agreement with statements about the severity COVID-19 and the public health consequences of the pandemic (e.g., *COVID-19 is no worse than the flu)* (α = .91 [38]).

#### Intimate partner violence and antisocial behavior

Participants were asked if they ever had a romantic relationship with the same partner that lasted longer than 3 months. If yes (*n* = 884), participants completed 13 items taken from the Controlling and Abusive Tactics-2 [43] and Conflict Tactics Scale-2 [44] that asked how frequently (*never*, *rare*, *occasional*, *common*, *frequent*) they had engaged in a variety of behaviors to spy on or control (e.g., search partner’s purse, wallet, or cell phone; withhold car keys or disable vehicle), threaten or insult, or physically harm (push, shove, slap, punch or hit, use a knife or gun) their current or most recent romantic partner (α = .97).

All participants also completed nine items that asked how many times (*never*, *once or twice*, *several times*, *many times*) they had engaged in various types of antisocial behavior including theft, destroying property, lying and conning, and aggression and violence, as well as being arrested and suspended or expelled from school (α = .93).

#### Mental health problems and substance use

We used the Suicidality (6-items; α = .93) and general Depression scales (20-items; α = .95) from the Inventory of Depression and Anxiety Symptoms (IDAS [45]) and the Generalized Anxiety Disorder-7 scale (GAD-7 [46]) (α = .94) to assess suicidal thoughts, self-harm behaviors, and overall depressive and non-specific anxiety symptoms over the past two weeks. We assessed recent alcohol use by calculating the mean of three items related to drinking alcohol in the past 30 days including average number of drinks per week, number of binge drinking episodes (i.e., five or more drinks on one occasion), and greatest number of drinks consumed in a 24-hour period (α = .87). We also assessed lifetime alcohol use problems using a 10-item scale that covered risky behaviors, negative consequences, lack of control, and tolerance associated with alcohol use (α = .95). Recent nicotine use was assessed using frequency of smoking cigarettes, using smokeless tobacco, or e-cigarettes in the past 30 days (*Never*, *once or twice*, *occasionally but not regularly*, *regularly*). The highest frequency reported among the three nicotine questions was used for the nicotine use variable.

#### Personality

We included six-items from the Desire for Power scale (α = .93 [47]) to assess desires to have influence, authority, power, and control over others. We used three items from the Belief in a Dangerous World scale (α = .82 [48]) to assess attitudes about an increasing degradation in the morals, order, and safety of society. The Big Five Inventory-2 short form (BFI-2-S; 30-items) was used to assess extraversion (sociability, assertiveness, energy level; α = .71), agreeableness (compassion, respectfulness, trust; α = .76), conscientiousness (organization, productiveness, responsibility, α = .78), negative emotionality (anxiety, depression, emotional volatility, α = .85), and open-mindedness (aesthetic sensitivity, intellectual curiosity, creative imagination, α = .68).

### Statistical analysis

The sample was divided into three groups: participants who reported purchasing a gun since March 2020 (*n* = 103; *COVID-19 pandemic gun buyers*), participants who reported owning a gun or rifle but not purchasing a gun since March 2020 (*n* = 170; *Pre-COVID-19 pandemic gun owners*), and participants who reported that they neither owned a gun nor purchased a gun since March 2020 (*n* = 763; *Non-gun owners*). We then compared the three groups on the outcome variables by fitting univariate ANOVAs and calculated Cohen’s *d* for the mean differences to provide an estimate of the effect sizes (*d* = 0.20 small effect, 0.50 medium effect, >0.80 large effect). Because there were large group differences for age and to a lesser degree for sex, we also fit ANCOVAs that included age and sex as covariates and report the adjusted partial η^2^ to provide a measure of the effect size associated with group membership after accounting for age and sex. We used an α = .005 as the threshold for statistical significance and a Bonferroni correction for multiple pairwise comparisons in post hoc analyses.

## Results

### Demographic comparisons

Table 1 reports the demographic comparisons of gun ownership groups. The COVID-19 pandemic gun buyer group had a much younger mean age than the non-gun owner (*d* = -0.85) and pre-COVID-19 pandemic gun owner groups (*d* = -0.97) [*F*(2, 1033) = 28.8, *p* < .001], with over half the pandemic gun buyers being between 30–39 years-old and 70% being under 40 years-old. The pandemic gun buyer (69.9%) and pre-pandemic gun owner groups (64.0%) included a significantly greater proportion of male participants than the non-gun owner group (45.3%). A higher proportion of the pandemic gun buyers (91.3%) reported White race than the non-gun owners (72.0%). Pandemic gun buyers reported higher mean income than the non-gun owners [*F*(2, 1033) = 10.9, *p* < .001] and had the highest proportion of members that reported an annual income of more than $100,000 (44.7% versus 26.6% for non-gun owners and 25.6% for pre-pandemic gun owners). The three groups did not differ in educational attainment or rates of Hispanic ethnicity.

**Table 1:** Demographic comparisons of gun ownership groups.

	Gun Ownership Groups			Group Comparisons		
**Variable**	COVID-19 Gun buyer (*n* = 103)	Pre-COVID-19 Gun owner (*n* = 170)	Non-gun owner (*n* = 763)	COVID-19 Gun buyer vs Pre-COVID-19 Gun owner	COVID-19 Gun buyer vs Non-gun owner	Pre-COVID-19 Gun owner vs Non-gun owner
**Sex** [% (*n*)]				χ^2^ (df)		
Female	30.1 (31)	35.9 (61)	54.8 (418)			
Male	69.9 (72)	64.1 (109)	45.2 (345)	1.0 (1)	**22.2 (1)**	**19.9 (1)**
**Race** [% (*n*)]						
White	91.3 (94)	81.2 (138)	72.0 (549)			
Black	6.8 (7)	13.3 (21)	15.1 (115)			
Asian	1.0 (1)	4.1 (7)	7.5 (57)			
Other	1.0 (1)	2.4 (4)	5.5 (42)	5.5 (3)	**18.5 (3)**	7.4 (3)
**Hispanic** [% (*n*)]						
Yes	21.4 (22)	11.2 (19)	18.1 (138)			
No	78.6 (81)	88.8 (151)	81.9 (625)	5.2 (1)	0.6 (1)	4.7 (1)
**Political Affiliation** [% (*n*)]						
Democratic	48.9 (44)	33.1 (53)	48.1 (351)			
Republican	40.0 (36)	38.8 (62)	19.9 (145)			
Independent or Unaffiliated	11.1 (10)	28.1 (45)	32.1 (234)	**11.3 (2)**	**26.5 (2)**	**27.2 (2)**
**Age (years)** [% (*n*)]					Cohen’s *d*	
18–29	19.4 (20)	9.3 (16)	12.7 (97)			
30–39	51.5 (53)	26.7 (46)	22.7 (173)			
40–49	18.4 (19)	12.2 (21)	17.6 (134)			
50–59	3.9 (4)	14.0 (24)	16.8 (128)			
60–69	3.9 (4)	25.6 (44)	21.5 (164)			
70+	2.9 (3)	12.2 (21)	8.8 (67)			
Mean (SD)	36.6 (11.1)	50.3 (16.5)	48.3 (15.9)	**-0.97**	**-0.85**	0.12
**Education** [% (*n*)]						
Less than high school	3.9 (4)	1.2 (2)	2.8 (21)			
High School Diploma	13.6 (14)	14.1 (24)	15.5 (118)			
Some college	19.4 (20)	31.2 (53)	26.5 (202)			
Bachelor’s degree	25.2 (26)	31.8 (54)	32.1 (245)			
Master’s degree	32.0 (33)	17.1 (29)	19.0 (145)			
Doctorate	5.8 (6)	4.7 (8)	4.2 (32)			
Mean (SD)	7.3 (2.0)	7.0 (1.8)	7.0 (1.9)	0.13	0.13	0.00
**Household Income** [% (*n*)]						
Less than $50,000	22.3 (23)	29.4 (50)	42.3 (323)			
$50,000-$99,999	33.0 (34)	44.7 (76)	31.1 (237)			
$100,000+	44.7 (46)	25.9 (44)	26.6 (203)			
Mean (SD)	5.0 (1.7)	4.5 (1.7)	4.1 (1.9)	0.22	**0.47**	0.21

Bold = *p* < .005. Statistical tests are χ^2^(df) for categorical variables, and Cohen’s *d* for quantitative variables with *p*-values Bonferroni adjusted for multiple pairwise comparisons in univariate ANOVAs.

The pandemic gun buyers and pre-pandemic gun owners both differed from non-gun owners in terms of political affiliation. Pandemic gun buyers and pre-pandemic gun owners were both more likely to affiliate with the Republican party than non-gun owners. Additionally, pre-pandemic gun owners were less likely to affiliate with the Democratic party, and pandemic gun buyers were less likely to be independent or unaffiliated than non-gun owners.

### Political attitudes

Table 2 provides means, standard deviations, sample sizes, and mean difference effect sizes (Cohen’s *d*s) across the outcome variables of interest. Test statistics (e.g., *F-* and *p*-values) for each ANOVA/ANCOVA model are provided in the supplemental tables. COVID-19 pandemic gun buyers reported much greater mean-levels of confidence and belief in QAnon information and conspiracies, and greater endorsement of pro-gun attitudes and Christian nationalism beliefs than non-gun owners (mean *d* = 1.47) and pre-COVID-19 pandemic gun owners (mean *d* = 1.19). Pandemic gun buyers also reported much greater mean-levels of skepticism about the seriousness of the COVID-19 pandemic, anti-vax beliefs, and comfort with engaging in activities in public places during the COVID-19 pandemic than non-gun owners (mean *d* = 0.94) and pre-pandemic gun owners (mean *d* = 0.86). Pandemic gun buyers also reported higher approval of past president Donald Trump than non-gun owners and pre-pandemic gun owners (mean *d* = 0.76), and higher approval of current president Joe Biden than pre-pandemic gun owners (*d* = 0.60). Pre-pandemic gun owners reported greater mean levels of pro-gun attitudes (*d* = 0.68), pro-police attitudes (*d* = 0.38), and lower approval of President Joe Biden (*d* = -0.41) than non-gun owners. All of these group differences were statistically significant (*p* < .005) after adjusting for age, sex, and multiple group comparisons.

**Table 2:** Gun ownership group comparisons on political attitudes, violence, mental health, and personality.

	Gun Ownership Group Mean (SD)			Group Comparison Cohen’s *d*			
**Variable**	COVID-19 Gun buyer (*n* = 103)	Pre-COVID-19 Gun owner (*n* = 170)	Non-gun owner (*n* = 763)	COVID-19 Gun buyer vs Pre-COVID-19 Gun owner	COVID-19 Gun buyer vs Non-gun owner	Pre-COVID-19 Gun owner vs Non-gun owner	Partial *η*^2^/ Partial *η*^2^ adj age & sex
**Political Attitudes**							
Q-Anon Beliefs	64.5 (8.7)	48.4 (8.7)	48.4 (8.8)	1.83*	1.83*	0.01	**.225**/ **.181**
Pro-Gun Attitudes	59.7 (5.8)	54.1 (8.9)	47.8 (9.6)	0.75*	1.50*	0.68*	**.158/ .133**
Christian Nationalism	58.1 (6.6)	49.8 (9.9)	49.0 (9.9)	0.99*	1.09*	0.09	**.074/ .081**
COVID-19 Skepticism	59.6 (7.9)	49.9 (9.6)	48.8 (9.7)	1.11*	1.22*	0.11	**.097**/ **.063**
Anti-Vax Beliefs	57.0 (6.8)	49.5 (10.4)	49.2 (9.9)	0.85*	0.92*	0.03	**.054/ .035**
COVID-19 Risk Estimates	55.9 (9.4)	49.7 (10.4)	49.3 (9.8)	0.62*	0.69*	0.04	**.038**/ **.023**
Trump Approval	57.1 (9.3)	50.8 (10.1)	48.8 (9.7)	0.65*	0.87*	0.20	**.061/ .048**
Biden Approval	52.3 (9.4)	46.4 (10.3)	50.5 (9.8)	0.60*	0.18	-0.41*	**.028/ .031**
Pro-Police Attitudes	51.1 (7.7)	53.2 (11.2)	49.1 (9.8)	-0.21	0.22	0.38*	**.023/ .021**
**IPV and Antisocial Behavior**							
Intimate Partner Violence	68.9 (16.8)	47.7 (5.6)	47.7 (5.2)	1.69*	1.71*	0.01	**.442/ .394**
Antisocial Behavior	66.2 (14.8)	49.4 (7.9)	48.0 (7.3)	1.41*	1.56*	0.20	**.293/ .249**
**Mental Health**							
IDAS Suicidality	67.8 (15.2)	48.8 (8.1)	47.9 (6.6)	1.56*	1.70*	0.12	**.350/ .302**
IDAS General Depression	62.5 (11.8)	49.9 (9.8)	48.3 (8.5)	1.16*	1.38*	0.17	**.176/ .142**
Generalized Anxiety Disorder	60.6 (10.7)	50.1 (10.0)	48.6 (9.0)	1.01*	1.21*	0.16	**.127/ .097**
Drinking Composite	60.8 (12.4)	50.4 (9.9)	48.4 (8.7)	0.93*	1.16*	0.21	**.134/ .097**
Alcohol Use Problems	63.8 (22.0)	48.8 (7.0)	48.4 (5.8)	0.92*	0.96*	0.06	**.207/ .180**
Nicotine use	62.2 (11.6)	49.5 (9.9)	48.5 (8.6)	1.18*	1.34*	0.11	**.164/ .121**
**Personality**							
Desire for Power	59.3 (7.9)	49.1 (9.8)	47.7 (9.1)	1.15*	1.36*	0.15	**.196/ .155**
Belief in a Dangerous World	56.8 (6.3)	50.5 (10.1)	49.0 (10.0)	0.75*	0.94*	0.15	**.054/ .053**
Agreeableness	42.8 (8.6)	50.2 (9.7)	51.5 (9.7)	-0.80*	-0.95*	-0.15	**.068/ .041**
Conscientiousness	44.4 (8.7)	52.3 (9.6)	52.1 (9.4)	-0.85*	-0.84*	0.02	**.057/ .029**
Openness	45.0 (6.8)	49.6 (10.4)	51.1 (10.1)	-0.52*	-0.71*	-0.15	**.033/ .036**
Negative Emotions	53.4 (6.5)	47.8 (9.9)	48.3 (10.0)	**0.67**	**0.60**	-0.05	**.025/** .009
Extraversion	48.9 (6.2)	51.1 (10.2)	50.4 (10.5)	-0.26	-0.18	0.07	.003/ .001

Bold = *p* < .005 Bonferroni adjusted for multiple pairwise comparisons

* *p* < .005 Bonferroni adjusted for multiple pairwise comparisons after adjusting for age and sex

### Intimate partner violence, antisocial behavior, mental health problems, and substance use

COVID-19 pandemic gun buyers reported much greater mean-levels of intimate partner violence, antisocial behavior, suicidality, depression, and generalized anxiety than non-gun owners (mean *d* = 1.51) and pre-COVID-19 pandemic gun owners (mean *d* = 1.37). Pandemic gun buyers also reported greater mean-levels of alcohol and nicotine use in the past 30 days and lifetime alcohol use problems than non-gun owners (mean *d* = 1.15) and pre-pandemic gun owners (mean *d* = 1.01). Differences between the pre-pandemic gun owners and non-gun owners on measures of intimate partner violence, antisocial behavior, mental health, and substance use were small (mean *d* = 0.13) and not statistically significant after adjusting for age, sex, and multiple group comparisons.

### Personality

COVID-19 pandemic gun buyers reported much greater mean levels of desire for power, beliefs in an increasingly dangerous and degenerating society, low agreeableness, low conscientiousness, low openness to experience, and negative emotions than non-gun owners (mean *d* = 0.90) and pre-COVID-19 pandemic gun owners (mean *d* = 0.79). Group differences on negative emotions were no longer statistically significant after adjusting for age and sex [*F*(2, 1006) = 4.6, *p* = .010]. Differences between the pre-pandemic gun owners and non-gun owners on the personality trait measures were small (mean *d* = 0.11) and not statistically significant after adjusting for age, sex, and multiple group comparisons.

### Characteristics of gun ownership

We also compared pre-COVID-19 pandemic gun owners and COVID-19 pandemic gun buyers on characteristics associated with gun ownership (Table 3). This information was not available for 25 participants who reported buying a gun during the COVID-19 pandemic, because they also reported that they do not currently own a gun. Pandemic gun buyers reported owning a greater number of handguns and total number of guns than pre-pandemic gun owners, and these differences were all statistically significant after adjusting for age and sex [*F*(1, 232) = 27.6, *p* < .001 for handguns and *F*(1, 228) = 19.4, *p* < .001 for total number of guns]. A greater proportion of pandemic gun buyers reported storing their firearm in a locked safe or cabinet than pre-pandemic gun owners, while a greater proportion of pre-pandemic gun owners reported their firearm was neither stored in a locked safe or cabinet and nor stored separately from ammunition than pandemic gun buyers. A greater proportion of pre-pandemic gun owners reported the primary reason for owning a gun was protection in the home, whereas a greater proportion of pandemic gun buyers reported the primary reason for owning a gun was hunting or sport or protection outside the home. A much larger proportion of pandemic gun buyers (74.4%) reported they carried a gun outside the home for reasons other than hunting than the pre-pandemic gun owners (25.6%). Relatedly, a greater proportion of pandemic gun buyers (80.8%) reported having a state issued permit to carry a firearm than pre-pandemic gun owners (46.5%).

**Table 3:** Characteristics of gun ownership.

	COVID-19 Gun buyer (*n* = 78)	Pre-COVID-19 Gun owner (*n* = 170)	Group Comparison Effect Size
**How many guns do you own?** [Mean (SD)]			Cohen’s *d*
Handguns	3.6 (5.2)	1.6 (1.3)	**0.52**
Rifles or long guns	3.2 (5.3)	1.7 (3.6)	0.32
Total guns	6.7 (10.1)	3.3 (4.1)	**0.44**
**How is your firearm stored?** [% (*n*)]			χ^2^(df)
Stored in a locked safe or cabinet	51.3 (40)	34.1 (58)	**12.8 (3)**
Stored separately from ammunition	26.9 (21)	25.9 (44)	
Both of the above	17.9 (14)	21.2 (36)	
None of the above	3.8 (3)	18.8 (32)	
**What is primary reason for owning a gun?** [% (*n*)]			
Hunting or sport	44.9 (35)	21.8 (37)	**23.1 (4)**
Protection in the home	33.3 (26)	63.5 (108)	
Protection outside the home	19.2 (15)	10.0 (17)	
Work	1.3 (1)	1.8 (3)	
Other	1.3 (1)	2.9 (5)	
**Do you carry a gun outside the home for reasons other than hunting?** [% (*n*)]			
Yes	74.4 (58)	25.6 (44)	**51.9 (1)**
No	25.6 (20)	74.1 (126)	
**Do you have a state issued permit to carry a firearm?** [% (*n*)]			
Yes	80.8 (63)	46.5 (79)	**26.1 (2)**
No	16.7 (13)	41.2 (70)	
Not required	2.6 (2)	12.2 (21)	

Note. df = degrees of freedom. Twenty-five COVID-19 gun buyers are missing data for these questions because they reported they did not currently own a gun at the time of the assessment completed between September 16, 2021 and October 11, 2021.

### Supplemental analysis

Because COVID-19 pandemic gun buyers reported owning more guns than pre-COVID-19 pandemic gun owners, we conducted supplemental analyses that included the total number of guns a participant owned as a covariate in the ANOVAs. After adjusting for the other variables in the model (group membership, age, and sex), number of guns owned was a statistically significant predictor (*p* < .005) of lower Biden approval and suicidality only. After adjusting for the number of guns owned, group membership remained a significant predictor in all the models. Therefore, the group differences on the various criterion variables cannot be attributed to the COVID-19 gun buyers owning more guns than the pre-COVID-19 gun owners and non-gun owners.

## Discussion

During the COVID-19 pandemic, there was a spike in both gun purchases and gun-related violence [1, 4]. While causal effects have yet to be established between the increased gun sales and gun-related violence, we examined the characteristics of people who acquired a gun during the COVID-19 pandemic as a proxy for the level of risk that may follow the pandemic gun purchase surge, with a special interest in determining if pandemic gun buyers exhibited known risk factors associated with violence.

In our survey, the single question of whether a person bought a gun during the COVID-19 pandemic identified an extreme group of people in terms of psychological characteristics including several risk-factors reliably associated with violence and self-harm. These group differences were notable in content, variety of outcomes, and magnitude of the effects. Most of the effect sizes for differences between pandemic gun buyers and both non-gun owners and pre-pandemic gun owners were large by conventional standards (*d* > .80) and several far exceeded this threshold. These differences were detected across multiple domains including political attitudes, behaviors, mental health problems, and personality traits. It is relatively rare in psychological research for a single behavioral item to exhibit such strong discriminating power for both the size and variety of differences. For example, it is unlikely that comparing people who did or did not purchase a car or major appliance during the COVID-19 pandemic would yield similar differences.

The largest differences were observed for relatively extreme attitudes and behaviors, specifically, QAnon beliefs, intimate partner violence, and suicidality. For example, 76% of pandemic gun buyers (versus 15% non-gun owners and pre-pandemic gun owners) endorsed the belief that the government, media, and financial worlds in the United States are controlled by a group of Satan-worshiping pedophiles who run a global child sex trafficking operation (QAnon); 56% occasionally to frequently punch or hit their partner (versus 1.6% of non-gun owners and 3% of pre-pandemic gun owners); 55% had thoughts of suicide (versus 6% of non-gun owners and 10% of pre-pandemic gun owners) and 64% cut or burned themselves on purpose (versus 4.4% of non-gun owners and pre-pandemic gun owners) in the last two weeks. Relative to the full sample, these characteristics have relatively low base-rates, and all are associated with violent beliefs or behaviors and self-harm. Pandemic gun buyers also exhibited extreme elevations on other mental health problems including general depression and anxiety, heavy substance use, and antisocial behavior. The combination of these mental health problems, history of antisocial and violent behaviors, and conspiratorial belief systems are all strong correlates of violence and self-harm behaviors and raise serious safety concerns if people with this psychological profile have access to firearms.

Differences between the pandemic gun buyers and the other groups was not attributable to pandemic gun buyers being an especially small group, as they constituted roughly 10% of the sample (*n* > 100), providing adequate statistical power for analyses. This 10% estimate is similar to the 6% estimate from two large surveys conducted in 2020, which covered a smaller time period for gun buying than our study [2, 3] and indicates that buying a gun during the pandemic, while not normative, was not rare.

Nonetheless, there was also heterogeneity in the political attitudes and beliefs of pandemic gun buyers. Although they seemed to lean toward a right-wing political orientation, this was not entirely consistent or uniform. On average, pandemic gun buyers held extreme pro-gun attitudes (much higher even than pre-pandemic gun owners), endorsed high approval of former president Donald Trump, supported Christian nationalism beliefs, were skeptical about the seriousness of COVID-19 and endorsed anti-vax attitudes. In contrast, pandemic gun buyers also had slightly positive approval ratings for President Biden and higher approval of Biden than pre-pandemic gun owners and did not endorse high pro-police attitudes. These findings are consistent with earlier surveys showing a bipartisan surge in gun-buying during the pandemic [1, 26]. Further, while the mean-levels of the mental health problems were extremely elevated in the pandemic gun buyer group, there were some members of this group that endorsed few or no such problems. These findings suggest that despite the distinctiveness of the pandemic gun buyer group on many dimensions, there is significant heterogeneity such that it is likely this group could be further refined to identify especially high-risk individuals.

Demographically, pandemic gun buyers were disproportionately male, White, and younger than non-gun owners and pre-pandemic gun owners. Gun owners in general are more likely to be male, but the proportion of White participants in the pandemic gun buyer group is greater than gun owners in general, which raises some questions as to the extent of any purported increase in gun purchases among non-White minorities and women during the pandemic [30]. Younger age and male sex are both associated with risk for violence and death by suicide, as White men die of suicide, usually with a gun, at higher proportions than other demographic groups [49]. The predominance of younger age groups among the pandemic gun buyers suggests there may be developmental influences or cohort effects contributing to pandemic gun purchases, although it is important to note that age per se did not account for group differences in psychological profiles of the groups as it was a covariate in the analyses. Pandemic gun buyers reported higher income which likely facilitated acquiring firearms, and they did not differ in education, which dispels any notions that gun acquisition during the pandemic was disproportionately a phenomenon among people of lower income and education.

How do our findings help explain increases in gun violence that began in 2020? At present, they do not. The greatest increase and highest rates of gun-related homicides in 2020 were among Black males aged 10–44 years and among people with lower income levels [5]. Given that the perpetrator and victim of a homicide typically share similar racial and socioeconomic characteristics, it seems unlikely then that pandemic gun buyers, who were predominately White and above average income (i.e., incongruous with most victims), will account for much of the increase in gun-related homicides. Direct analyses, however, are needed to estimate the effect, if any, of the pandemic-era gun purchases on the increase in gun-related violence. However, it is important to track the *long-term* effects of the COVID-19 pandemic era gun purchases, especially since it is predicted that rates of gun violence will remain at relatively high levels through 2023. Most gun deaths are by suicide [49], and if pandemic gun buyers are more likely to have mental health and substance use problems, propensities toward aggression and self-harm, and beliefs that the world is dangerous, as we found, then we may expect to see more suicide deaths by guns from those gun buyers in the future.

Another key finding was that we detected few differences between non-gun owners and pre-pandemic gun owners on the many variables we examined. Pre-pandemic gun owners were most distinguished by greater pro-gun attitudes, though this difference was smaller than the difference between pre-pandemic gun owners and pandemic gun buyers (i.e., non-gun owners < pre-pandemic gun owners < pandemic gun buyers). Relative to non-gun owners, pre-pandemic gun owners were more likely to be male, affiliated with the Republican party, and endorsed higher pro-police attitudes and lower approval of President Biden. Pre-pandemic gun owners did not differ from non-gun-owners in mental health problems, substance use, antisocial behavior, intimate partner violence, personality traits, COVID-19 related variables, Trump approval, Christian nationalism, or QAnon beliefs. Pre-pandemic gun owners then may be slightly more conservative than non-gun owners, but as a group they did not endorse far-right political beliefs, nor did they exhibit elevations in mental health problems and antisocial behavior that are risk factors for violence and self-harm. This suggests that gun ownership *per se* is not indicative of mental health problems, antisocial behavior, and holding extreme political beliefs. Gun owners then are a diverse group, and it is especially interesting that purchasing a gun during the COVID-19 pandemic was so effective at identifying a group of gun owners with such elevated levels of risk factors for violence and self-harm.

This study provides a broad picture of the distinct characteristics of pandemic gun buyers, which raises important questions about future risk of violence or self-harm with guns. Nonetheless, the study had some important limitations. One is that nearly a quarter of pandemic gun buyers did not report owning a gun. We did not ask pandemic gun buyers if they still owned the gun(s) they recently purchased or disposed of them in some way, so the nature of this discrepancy is unclear. It is possible the discrepancy is due to careless or invalid responding but removing those individuals did not change the results or effect sizes (if anything they were slightly larger). That is, the pandemic buyers who reported not owning a gun exhibited a nearly identical–though not quite as extreme–pattern of elevations in mental health problems, political attitudes, personality, etc., to pandemic gun buyers who reported currently owning a gun. It may be that pandemic gun buyers who did not report owning a gun later returned the gun after the purchase or disposed of the gun in some other way. We hope to clarify the reason for this discrepancy by making edits to the survey during the next round of data collection. Another limitation is the potential for low reliability and poor validity for self-reports in a survey. We attempted to reduce the impact of such potential influences by including attention checks during the survey and excluding participants with illogical, inconsistent, and anomalous (e.g., straight-lining) response patterns. Further, the pattern of correlations among the measures are generally consistent with the theoretical models of the given constructs, and a prior report that examined our survey data for waves 1 thru 5 typically found rank-order stability of *r* > .75 over a three-month test-retest period for measures of trait constructs, indicative of relatively high reliability [50].

Several other analyses could also be performed for future directions. For example, geographical information and neighborhood characteristics (e.g., urban vs rural, crime rate) might also distinguish these groups, as well as state and local ordinances regarding gun ownership. Also, more questions could be asked regarding the specific motivations for owning a gun and acquiring a gun during the COVID-19 pandemic. For example, person-centered analyses (e.g., cluster analysis, latent profile analysis) might be effective in further parsing the pandemic gun buyer group and gun owners in general. Finally, given that this study only describes the different gun ownership groups, we are unable to make any inferences as to any causal influences on gun buying during the COVID-19 pandemic or potential group differences in gun violence.

Despite these limitations, these analyses using a large and fairly representative sample of adults in the United States highlight the potential implications of gun sales during the COVID-19 pandemic. Although state and federal firearm policies are important determinants of gun deaths [12], more investigations can take a closer look at person-level characteristics of gun owners which can exacerbate risk of gun deaths or injuries. Behavioral health research plays a key role in public health, and in the wake of the 2020 rise in gun sales and deaths, our data can help inform public health campaigns to reach the pandemic era gun buyers who are at the highest risk of using their firearms in destructive ways.

## Supporting information

S1 File (DOCX)
