# Supplementary material for: Who bought a gun during the COVID-19 pandemic in the United States?: Associations with QAnon beliefs, right-wing political attitudes, intimate partner violence, antisocial behavior, suicidality, and mental health and substance use problems
Source: PLoS One. 2023 Aug 29;18(8):e0290770. doi: 10.1371/journal.pone.0290770 (PMC10464976; doi:10.1371/journal.pone.0290770)
Supplement: S1 File — (DOCX) [file pone.0290770.s001.docx]

**Supplemental Table 1. Demographic comparisons of gun ownership groups**

|  | Gun Ownership Groups | | | Group Comparisons | | |
| --- | --- | --- | --- | --- | --- | --- |
|  | 1. COVID-19 Gun buyer  (*n* = 103) | 2. Pre-COVID-19 Gun owner  (*n* =170) | 3. Non-gun owner  (*n* = 763) | 1 vs 2 | 1 vs 3 | 2 vs 3 |
| **Sex** [% (*n*)] |  |  |  |  |  |  |
| Female | 30.1 (31) | 35.9 (61) | 54.8 (418) |  |  |  |
| Male | 69.9 (72) | 64.1 (109) | 45.2 (345) | χ^2^(1) = 1.0, *p* = .33 | **χ^2^(1) = 22.2, *p* < .001** | **χ^2^(1) = 19.9, *p* < .001** |
| **Race** [% (*n*)] |  |  |  |  |  |  |
| White | 91.3 (94) | 81.2 (138) | 72.0 (549) |  |  |  |
| Black | 6.8 (7) | 13.3 (21) | 15.1 (115) |  |  |  |
| Asian | 1.0 (1) | 4.1 (7) | 7.5 (57) |  |  |  |
| Other | 1.0 (1) | 2.4 (4) | 5.5 (42) | χ^2^(3) = 5.5, *p* = .137 | **χ^2^(3) = 18.5, *p* < .001** | χ^2^(3) = 7.4, *p* = .060 |
| **Hispanic** [% (*n*)] |  |  |  |  |  |  |
| Yes | 21.4 (22) | 11.2 (19) | 18.1 (138) |  |  |  |
| No | 78.6 (81) | 88.8 (151) | 81.9 (625) | χ^2^(1) = 5.2, *p* = .022 | χ^2^(1) = 0.6, *p* = .422 | χ^2^(1) = 4.7, *p* = .029 |
| **Political Affiliation** [% (*n*)] |  |  |  |  |  |  |
| Democratic | 48.9 (44) | 33.1 (53) | 48.1 (351) |  |  |  |
| Republican | 40.0 (36) | 38.8 (62) | 19.9 (145) |  |  |  |
| Independent or Unaffiliated | 11.1 (10) | 28.1 (45) | 32.1 (234) | **χ^2^(2) = 11.3, *p* = .004** | **χ^2^(2) = 26.5, *p* < .001** | **χ^2^(2) = 27.2, *p* < .001** |
| **Age (years)** [% (*n*)] |  |  |  |  |  |  |
| 18-29 | 19.4 (20) | 9.3 (16) | 12.7 (97) |  |  |  |
| 30-39 | 51.5 (53) | 26.7 (46) | 22.7 (173) |  |  |  |
| 40-49 | 18.4 (19) | 12.2 (21) | 17.6 (134) |  |  |  |
| 50-59 | 3.9 (4) | 14.0 (24) | 16.8 (128) |  |  |  |
| 60-69 | 3.9 (4) | 25.6 (44) | 21.5 (164) |  |  |  |
| 70+ | 2.9 (3) | 12.2 (21) | 8.8 (67) |  |  |  |
| Mean (SD) | 36.6 (11.1) | 50.3 (16.5) | 48.3 (15.9) | ***t*(271) = 7.44, *p* < .001** | ***t*(864) = 7.22, *p* < . 001** | *t*(931) = 1.47, *p* = .141 |
| *F-*statistic |  |  |  | ***F*(2, 1033) = 28.8, *p* < .001** | | |
| Cohen’s *d* |  |  |  | ***d* = -0.97** | ***d* = -0.85** | *d* = 0.12 |
| **Education** [% (*n*)] |  |  |  |  |  |  |
| Less than high school | 3.9 (4) | 1.2 (2) | 2.8 (21) |  |  |  |
| High School Diploma | 13.6 (14) | 14.1 (24) | 15.5 (118) |  |  |  |
| Some college | 19.4 (20) | 31.2 (53) | 26.5 (202) |  |  |  |
| Bachelor’s degree | 25.2 (26) | 31.8 (54) | 32.1 (245) |  |  |  |
| Master’s degree | 32.0 (33) | 17.1 (29) | 19.0 (145) |  |  |  |
| Doctorate | 5.8 (6) | 4.7 (8) | 4.2 (32) |  |  |  |
| Mean (SD) | 7.3 (2.0) | 7.0 (1.8) | 7.0 (1.9) | *t*(271) = 1.09, *p* = .276 | *t(*864) = 1.31, *p* = .190 | *t*(931) = 0.28, *p* = .978 |
| *F-*statistic |  |  |  | *F*(2, 1033) = 0.9, *p* = .412 | | |
| Cohen’s *d* |  |  |  | *d* = 0.13 | *d* = 0.13 | *d* = 0.00 |
| **Household Income** [% (*n*)] |  |  |  |  |  |  |
| Less than $50,000 | 22.3 (23) | 29.4 (50) | 42.3 (323) |  |  |  |
| $50,000-$99,999 | 33.0 (34) | 44.7 (76) | 31.1 (237) |  |  |  |
| $100,000+ | 44.7 (46) | 25.9 (44) | 26.6 (203) |  |  |  |
| Mean (SD) | 5.0 (1.7) | 4.5 (1.7) | 4.1 (1.9) | *t*(271) = 2.13, *p* = .034 | ***t*(864) = 4.22, *p* < . 001** | *t*(931) = 2.43, *p* = .015 |
| *F-*statistic |  |  |  | ***F*(2, 1033) = 10.9, *p* < .001** | | |
| Cohen’s *d* |  |  |  | *d* = 0.22 | ***d* = 0.47** | *d* = 0.21 |

Bold = *p* < .005. Cohen’s *d* for quantitative variables include *p*-values Bonferroni adjusted for multiple pairwise comparisons in univariate ANOVAs.

**Supplemental Table 2. Unadjusted and adjusted (age, sex) group comparisons on political attitudes, violence, mental health, and personality.**

|  | Gun Ownership groups | | | Group Comparisons Cohen’s *d* | | | ANOVA/ANCOVA  Test statistics | |
| --- | --- | --- | --- | --- | --- | --- | --- | --- |
|  | 1. COVID-19 Gun buyer | 2. Pre-COVID-19 Gun owner | 3. Non-gun owner | 1 vs 2 | 1 vs 3 | 2 vs 3 | *F*-value, *p*-value | Partial *η*^2^ |
| **Political Attitudes** |  |  |  |  |  |  |  |  |
| Q-Anon Beliefs | 64.5 (8.7) | 48.4 (8.7) | 48.4 (8.8) | **1.83*** | **1.83*** | 0.01 | ***F*(2, 1033) = 148.8,**  ***p* < .001** | **.225** |
| adj age, sex |  |  |  |  |  |  | ***F*(2, 1031) = 113.7,**  ***p* < .001** | **.181** |
| Pro-Gun Attitudes | 59.7 (5.8) | 54.1 (8.9) | 47.8 (9.6) | **0.75*** | **1.50*** | **0.68*** | ***F*(2, 1033) = 96.7,**  ***p* < .001** | **.158** |
| adj age, sex |  |  |  |  |  |  | ***F*(2, 1031) = 78.9,**  ***p* < .001** | **.133** |
| Christian Nationalism | 58.1 (6.6) | 49.8 (9.9) | 49.0 (9.9) | **0.99*** | **1.09*** | 0.09 | ***F*(2, 1033) = 41.2,**  ***p* < .001** | **.074** |
| adj age, sex |  |  |  |  |  |  | ***F*(2, 1031) = 45.6,**  ***p* < .001** | **.081** |
| COVID-19 Skepticism | 59.6 (7.9) | 49.9 (9.6) | 48.8 (9.7) | **1.11*** | **1.22*** | 0.11 | ***F*(2, 1018) = 54.6,**  ***p* < .001** | **.097** |
| adj age, sex |  |  |  |  |  |  | ***F*(2, 1016) = 34.0,**  ***p* < .001** | **.063** |
| Anti-Vax Beliefs | 57.0 (6.8) | 49.5 (10.4) | 49.2 (9.9) | **0.85*** | **0.92*** | 0.03 | ***F*(2, 1015) = 28.7,**  ***p* < .001** | **.054** |
| adj age, sex |  |  |  |  |  |  | ***F*(2, 1013) = 18.4,**  ***p* < .001** | **.035** |
| COVID-19 Risk Estimates | 55.9 (9.4) | 49.7 (10.4) | 49.3 (9.8) | **0.62*** | **0.69*** | 0.04 | ***F*(2, 1033) = 20.6,**  ***p* < .001** | **.038** |
| adj age, sex |  |  |  |  |  |  | ***F*(2, 1031) = 12.4,**  ***p* < .001** | **.023** |
| Trump Approval | 57.1 (9.3) | 50.8 (10.1) | 48.8 (9.7) | **0.65*** | **0.87*** | 0.20 | ***F*(2, 1033) = 33.4,**  ***p* < .001** | **.061** |
| adj age, sex |  |  |  |  |  |  | ***F*(2, 1031) = 26.0,**  ***p* < .001** | **.048** |
| Biden Approval | 52.3 (9.4) | 46.4 (10.3) | 50.5 (9.8) | **0.60*** | 0.18 | **-0.41*** | ***F*(2, 1018) = 14.9,**  ***p* < .001** | **.028** |
| adj age, sex |  |  |  |  |  |  | ***F*(2, 1016) = 16.5,**  ***p* < .001** | **.031** |
| Pro-Police Attitudes | 51.1 (7.7) | 53.2 (11.2) | 49.1 (9.8) | -0.21 | 0.22 | **0.38*** | ***F*(2, 1033) = 12.2,**  ***p* < .001** | **.023** |
| adj age, sex |  |  |  |  |  |  | ***F*(2, 1031) = 10.9,**  ***p* < .001** | **.021** |
| **IPV and Antisocial Behavior** |  |  |  |  |  |  |  |  |
| Intimate Partner Violence | 68.9 (16.8) | 47.7 (5.6) | 47.7 (5.2) | **1.69*** | **1.71*** | 0.01 | ***F*(2, 879) = 348.4,**  ***p* < .001** | **.442** |
| adj age, sex |  |  |  |  |  |  | ***F*(2, 877) = 285.1,**  ***p* < .001** | **.394** |
| Antisocial Behavior | 66.2 (14.8) | 49.4 (7.9) | 48.0 (7.3) | **1.41*** | **1.56*** | 0.20 | ***F*(2, 1033) = 214.1,**  ***p* < .001** | **.293** |
| adj age, sex |  |  |  |  |  |  | ***F*(2, 1031) = 171.0,**  ***p* < .001** | **.249** |
| **Mental Health** |  |  |  |  |  |  |  |  |
| IDAS Suicidality | 67.8 (15.2) | 48.8 (8.1) | 47.9 (6.6) | **1.56*** | **1.70*** | 0.12 | ***F*(2, 960) = 258.3,**  ***p* < .001** | **.350** |
| adj age, sex |  |  |  |  |  |  | ***F*(2, 958) = 207.5,**  ***p* < .001** | **.302** |
| IDAS General Depression | 62.5 (11.8) | 49.9 (9.8) | 48.3 (8.5) | **1.16*** | **1.38*** | 0.17 | ***F*(2, 960) = 102.6,**  ***p* < .001** | **.176** |
| adj age, sex |  |  |  |  |  |  | ***F*(2, 958) = 79.4,**  ***p* < .001** | **.142** |
| Generalized Anxiety Disorder | 60.6 (10.7) | 50.1 (10.0) | 48.6 (9.0) | **1.01*** | **1.21*** | 0.16 | ***F*(2, 960) = 69.6,**  ***p* < .001** | **.127** |
| adj age, sex |  |  |  |  |  |  | ***F*(2, 958) = 51.7,**  ***p* < .001** | **.097** |
| Drinking Composite | 60.8 (12.4) | 50.4 (9.9) | 48.4 (8.7) | **0.93*** | **1.16*** | 0.21 | ***F*(2, 1033) = 79.9,**  ***p* < .001** | **.134** |
| adj age, sex |  |  |  |  |  |  | ***F*(2, 1031) = 55.4,**  ***p* < .001** | **.097** |
| Alcohol Use Problems | 63.8 (22.0) | 48.8 (7.0) | 48.4 (5.8) | **0.92*** | **0.96*** | 0.06 | ***F*(2, 1033) = 134.9,**  ***p* < .001** | **.207** |
| adj age, sex |  |  |  |  |  |  | ***F*(2, 1031) = 113.1,**  ***p* < .001** | **.180** |
| Nicotine use | 62.2 (11.6) | 49.5 (9.9) | 48.5 (8.6) | **1.18*** | **1.34*** | 0.11 | ***F*(2, 1033) = 101.3,**  ***p* < .001** | **.164** |
| adj age, sex |  |  |  |  |  |  | ***F*(2, 1031) = 71.1,**  ***p* < .001** | **.121** |
| **Personality** |  |  |  |  |  |  |  |  |
| Desire for Power | 59.3 (7.9) | 49.1 (9.8) | 47.7 (9.1) | **1.15*** | **1.36*** | 0.15 | ***F*(2, 444) = 54.0,**  ***p* < .001** | **.196** |
| adj age, sex |  |  |  |  |  |  | ***F*(2, 442) = 40.5,**  ***p* < .001** | **.155** |
| Belief in a Dangerous World | 56.8 (6.3) | 50.5 (10.1) | 49.0 (10.0) | **0.75*** | **0.94*** | 0.15 | ***F*(2, 1033) = 29.6,**  ***p* < .001** | **.054** |
| adj age, sex |  |  |  |  |  |  | ***F*(2, 1031) = 28.8,**  ***p* < .001** | **.053** |
| Agreeableness | 42.8 (8.6) | 50.2 (9.7) | 51.5 (9.7) | **-0.80*** | **-0.95*** | -0.15 | ***F*(2, 1006) = 36.9,**  ***p* < .001** | **.068** |
| adj age, sex |  |  |  |  |  |  | ***F*(2, 1004) = 21.2,**  ***p* < .001** | **.041** |
| Conscientiousness | 44.4 (8.7) | 52.3 (9.6) | 52.1 (9.4) | **-0.85*** | **-0.84*** | 0.02 | ***F*(2, 1006) = 30.2,**  ***p* < .001** | **.057** |
| adj age, sex |  |  |  |  |  |  | ***F*(2, 1004) = 15.2,**  ***p* < .001** | **.029** |
| Openness | 45.0 (6.8) | 49.6 (10.4) | 51.1 (10.1) | **-0.52*** | **-0.71*** | -0.15 | ***F*(2, 1006) = 17.4,**  ***p* < .001** | **.033** |
| adj age, sex |  |  |  |  |  |  | ***F*(2, 1004) = 18.8,**  ***p* < .001** | **.036** |
| Negative Emotions | 53.4 (6.5) | 47.8 (9.9) | 48.3 (10.0) | **0.67** | **0.60** | -0.05 | ***F*(2, 1006) = 12.9,**  ***p* < .001** | **.025** |
| adj age, sex |  |  |  |  |  |  | *F*(2, 1004) = 4.6,  *p* = .010 | .009 |
| Extraversion | 48.9 (6.2) | 51.1 (10.2) | 50.4 (10.5) | -0.26 | -0.18 | 0.07 | *F*(2, 1006) = 1.5,  *p* = .218 | .003 |
| adj age, sex |  |  |  |  |  |  | *F*(2, 1004) = 0.6,  *p* = .525 | .001 |

Bold = *p* < .005. Bold for group comparisons included Bonferroni adjusted *p*-values for multiple pairwise comparisons; * *p* < .005 Bonferroni adjusted for multiple pairwise comparisons after adjusting for age and sex in ANCOVA models. Adj = adjusted for age and sex in ANCOVA models

**Supplemental Table 3. Characteristics of gun ownership.**

|  | COVID-19  Gun buyer  (*n* = 78) | Pre-COVID-19  Gun owner  (*n* = 170) | Test Statistics | Effect Size  Cohen’s *d* |
| --- | --- | --- | --- | --- |
| **How many guns do you own?** [Mean (SD)] |  |  | *t*-statistics |  |
| Handguns | 3.6 (5.2) | 1.6 (1.3) | ***t*(234) = 4.6, *p* < .001** | **0.52** |
| Rifles or long guns | 3.2 (5.3) | 1.7 (3.6) | *t*(230) = 2.4, *p =* .017 | 0.32 |
| Total guns | 6.7 (10.1) | 3.3 (4.1) | ***t*(230) = 3.6, *p* < .001** | **0.44** |
| **How is your firearm stored?** [% (*n*)] |  |  | Chi square |  |
| Stored in a locked safe or cabinet | 51.3 (40) | 34.1 (58) | **χ^2^(3) = 12.8, *p* = .005** |  |
| Stored separately from ammunition | 26.9 (21) | 25.9 (44) |  |  |
| Both of the above | 17.9 (14) | 21.2 (36) |  |  |
| None of the above | 3.8 (3) | 18.8 (32) |  |  |
| **What is primary reason for owning a gun?** [% (*n*)] |  |  |  |  |
| Hunting or sport | 44.9 (35) | 21.8 (37) | **χ^2^(4) = 23.1, *p* < .001** |  |
| Protection in the home | 33.3 (26) | 63.5 (108) |  |  |
| Protection outside the home | 19.2 (15) | 10.0 (17) |  |  |
| Work | 1.3 (1) | 1.8 (3) |  |  |
| Other | 1.3 (1) | 2.9 (5) |  |  |
| **Do you carry a gun outside the home for reasons other than hunting?** [% (*n*)] |  |  |  |  |
| Yes | 74.4 (58) | 25.6 (44) | **χ^2^(1) = 51.9, *p* < .001** |  |
| No | 25.6 (20) | 74.1 (126) |  |  |
| **Do you have a state issued permit to carry a firearm?** [% (*n*)] |  |  |  |  |
| Yes | 80.8 (63) | 46.5 (79) | **χ^2^(2) = 26.1, *p* < .001** |  |
| No | 16.7 (13) | 41.2 (70) |  |  |
| Not required | 2.6 (2) | 12.2 (21) |  |  |

Note. Twenty-five COVID-19 gun buyers are missing data for these questions because they reported they did not currently own a gun at the time of the assessment completed between September 16, 2021 and October 11, 2021.
